# Serum heat shock protein 47 levels in patients with drug-induced lung disease

**DOI:** 10.1186/1465-9921-14-133

**Published:** 2013-11-20

**Authors:** Tomoyuki Kakugawa, Shin-ichi Yokota, Yuji Ishimatsu, Tomayoshi Hayashi, Shota Nakashima, Shintaro Hara, Noriho Sakamoto, Yasuhiro Matsuoka, Hiroshi Kubota, Mariko Mine, Hiroshi Mukae, Kazuhiro Nagata, Shigeru Kohno

**Affiliations:** 1 Second Department of Internal Medicine, Nagasaki University School of Medicine, Nagasaki, Japan; 2 Department of Microbiology, Sapporo Medical University School of Medicine, Sapporo, Japan; 3 Department of Pathology, Nagasaki University Hospital, Nagasaki, Japan; 4 Department of Molecular and Cellular Biology, Institute for Frontier Medical Sciences, Kyoto University, Kyoto, Japan; 5 Department of Life Science, Faculty and Graduate School of Engineering and Resource Science, Akita University, Akita, Japan; 6 Biostatistics Section, Division of Scientific Data Registry, Atomic Bomb Disease Institute, Nagasaki University, Nagasaki, Japan; 7 Department of Respiratory Medicine, School of Medicine, University of Occupational and Environmental Health, Kitakyushu, Japan; 8 Laboratory of Molecular and Cellular Biology, Faculty of Life Sciences, Kyoto Sangyo University, Kyoto, Japan

**Keywords:** Drug-induced lung disease, Krebs von den Lungen-6, Serum marker, Surfactant protein A, Surfactant protein D

## Abstract

**Background:**

Heat shock protein (HSP) 47 is a collagen-specific molecular chaperone that is required for molecular maturation of various types of collagens. We recently reported that HSP47 serum levels were markedly higher in patients with acute exacerbations of idiopathic pulmonary fibrosis (IPF) when compared with patients with stable IPF, suggesting that serum HSP47 levels correlate with interstitial pneumonia activity. The aim of this study was to evaluate serum HSP47 levels in patients with drug-induced lung disease (DILD).

**Methods:**

Findings from high-resolution computed tomographic chest scans of 47 patients with DILD were classified into one of four predominant patterns: organizing pneumonia (OP) (n = 4), nonspecific interstitial pneumonia (NSIP) (n = 24), hypersensitivity pneumonitis (HP) (n = 11), and diffuse alveolar damage (DAD) (n = 8). Serum levels of HSP47, Krebs von den Lungen-6 (KL-6), surfactant protein (SP)-A, and SP-D were measured in these patients.

**Results:**

The PaO_2_/fraction of inspired oxygen (FiO_2_) (P/F) ratios were significantly lower and the alveolar-arterial difference of oxygen (A-a DO_2_) was significantly higher in the DAD group than in the other groups. Patients with DAD had the worst outcomes among the different subgroups. Patients in the DAD group had significantly higher serum HSP47 levels than those in other groups. Receiver operating characteristic curves revealed that HSP47 was superior to KL-6, SP-A, and SP-D for discriminating between the DAD group and the other groups. The cut-off level for HSP47 that resulted in the highest diagnostic accuracy was 1711.5 pg/mL. The sensitivity, specificity, and diagnostic accuracy were 87.5%, 97.4%, and 95.7%, respectively. Serum levels of HSP47 in the group of patients requiring glucocorticoids were significantly higher than those in patients who experienced clinical improvement without glucocorticoid administration. Serum HSP47 levels also significantly correlated with various respiratory parameters.

**Conclusion:**

This study demonstrated that serum HSP47 levels were elevated in patients with DILD with a DAD pattern who had the worst outcomes among the different subgroups, and that this was correlated with P/F ratio and A-a DO_2_.

## Background

The increasing use of novel drugs such as tyrosine kinase inhibitors and disease-modifying anti-rheumatic drugs has resulted in an increased incidence of drug-induced lung disease (DILD). While DILD can progress rapidly and result in fatal outcomes even when the causative drugs are ceased promptly, there is currently insufficient data to help guide clinicians in terms of evaluating DILD severity, identifying patients at high-risk of poor outcomes, and selecting which patients might benefit from glucocorticoid administration.

Drugs can induce a variety of pathological reactions in the lung [1], with patients developing diffuse alveolar damage (DAD) having the worst outcomes. High-resolution computed tomographic (HRCT) chest scans are currently the best noninvasive method for predicting the underlying histologic pattern [1]. In one report, radiographic appearance was assessed in 70 patients with confirmed gefitinib-induced lung toxicity [2]; the mortality rate was significantly higher in patients with a pattern of extensive bilateral ground-glass attenuation or airspace consolidations with traction bronchiectasis in HRCT chest scans (a finding that is thought to reflect DAD) when compared with patients with other patterns of lung injury [2]. This finding is consistent with observations by Ichikado et al., who reported that traction bronchiectasis is an important prognostic CT finding in patients with acute interstitial pneumonia [3]. However, HRCT scanning is expensive, and the associated radiation exposure is associated with an increased risk of long-term complications. Therefore, the identification of other noninvasive markers that specifically identify DAD and correlate with respiratory status, disease severity, and outcomes would be of benefit.

A number of serum markers correlate with aspects of interstitial lung disease, including surfactant protein (SP)-A, SP-D, and Krebs von den Lungen-6 (KL-6), which is a circulating high-molecular weight glycoprotein expressed by type II pneumocytes [4,5]. Previous studies suggested that KL-6 is a useful diagnostic biomarker for DILD [6,7]. However, the clinical role of these serum markers in the diagnosis of DILD remains unclear. Moreover, there have not been any reports regarding noninvasive markers that specifically identify DAD and correlate with respiratory status, disease severity, and outcomes in patients with DILD.

Heat shock protein (HSP) 47 is a molecular chaperone that is required for molecular maturation of various types of collagens [8-11]. Increased levels of HSP47 in fibrotic diseases might lead to excessive assembly and intracellular processing of procollagen molecules, thereby contributing to the formation of fibrotic lesions [12]. Indeed, studies have shown that collagen accumulation and disease progression are associated with HSP47 protein levels in an experimental pulmonary fibrosis model [13-16]. Previous studies have also shown a close association between increased expression of HSP47 in fibrotic lung tissue and interstitial pneumonia activity [17-19].

We recently reported that HSP47 serum levels were markedly higher in patients with acute exacerbations of idiopathic pulmonary fibrosis (IPF) when compared with patients with stable IPF. Further, serum HSP47 levels are superior to those of KL-6, SP-A, and SP-D for discriminating between acute exacerbations of IPF and stable IPF [19]. These findings suggest that serum HSP47 levels correlate with interstitial pneumonia activity. Therefore, the goal of this study was to investigate the novel hypothesis that serum HSP47 might also correlate with DILD status.

## Materials and methods

### Study population

This was a retrospective study. Study subjects consisted of 47 patients who were admitted to Nagasaki University Hospital from January 1999 to March 2013. The diagnoses were based on a history of drug exposure, radiologic findings consistent with DILD, and exclusion of other common causes of pulmonary injury, such as opportunistic infection, radiation pneumonitis, pulmonary thromboembolism, oxygen administration, and progression of the primary illness [1,20]. The evidence for lung damage and exclusion of other causes were obtained by HRCT chest scanning and other methods including bronchoalveolar lavage (BAL) (n = 40) and transbronchial lung biopsy (n = 22). The timing of the HRCT chest scanning and BAL was determined by each attending physician based on the patients’ status. Sera were obtained at the time of diagnosis based on these clinical observations and stored at −80°C for later analysis. Causative drugs were determined according to history of drug exposure and response to withdrawal of the implicated drug, in addition to a lymphocyte stimulation test (n = 28). Data regarding patient characteristics were collected from clinical notes recorded at the time of diagnosis and included age, sex, smoking history, PaO_2_/fraction of inspired oxygen (FiO_2_) ratio (P/F ratio), and alveolar-arterial difference of oxygen (A-a DO_2_). Data regarding serum concentrations of KL-6, SP-A, and SP-D were also collected from the clinical notes recorded at the time of diagnosis. For records that lacked the data for these markers, measurements were performed using the preserved serum samples. Data regarding these markers were not obtained in some patients in which the volume of preserved serum samples was not sufficient. The 30-day mortality rates were determined for all disease groups. Sera were also obtained from healthy volunteers (14 men and 12 women; median age, 31 years; range, 25 to 59 years), all of whom had normal chest radiographs, were free of symptoms, and were not taking any medications.

The study protocol was approved by the Institutional Review Board of Nagasaki University Hospital and the Ethics Committee, Nagasaki University Graduate School of Biomedical Sciences. Written informed consent was obtained from all subjects.

### Subclassification of DILD based on HRCT findings

All patients underwent chest radiography and HRCT chest scanning. The HRCT findings were reviewed separately in random order by two independent observers who were not aware of the patients’ profiles and were categorized into four previously established patterns [1,2,7]: (1) organizing pneumonia (OP) pattern, showing peribronchial or subpleural consolidation or ground-glass opacities without fibrosis; (2) nonspecific interstitial pneumonia (NSIP) pattern, characterized by patchy or diffuse ground-glass opacities with associated reticular opacities, traction bronchiectasis, and bronchiolectasis; (3) hypersensitivity pneumonitis (HP) pattern, with diffuse ground glass opacities without fibrosis; and (4) DAD pattern, characterized by extensive bilateral ground glass attenuation and/or airspace consolidations with traction bronchiectasis and/or traction bronchiolectasis. Following the initial independent evaluations, divergent observations were resolved by consensus after consultation between the two observers.

### BAL and cell preparation

Bronchoscopy and BAL were performed as described previously [21].

### Sandwich enzyme-linked immunosorbent assay (ELISA) for determination of HSP47 concentration

Sandwich ELISA was performed to determine HSP47 concentration as described previously [22].

### Measurement of serum KL-6, SP-A, and SP-D levels

Serum levels of KL-6, SP-A, and SP-D were measured as described previously [19].

### Immunohistochemistry

Immunohistochemistry for HSP47 and type I procollagen was performed as described previously [17].

### Statistical analysis

The interobserver agreement was assessed using Kappa statistics [23]. Kappa values greater than 0.61 were considered to indicate good agreement between observers [23]. Continuous variables are expressed as medians (range). Differences among groups were determined by analysis of variance or by the Kruskal-Wallis test for continuous variables and the χ^2^ test for categorical variables, as appropriate. If a significant difference was found by analysis of variance, a pair-wise comparison was performed using the Scheffe method. Comparisons between two groups were made using the Wilcoxon two-sample test. Correlations between variables were assessed using Spearman nonparametric analysis. The upper left corner coordinate point of the receiver operating characteristic curve was used to determine the optimum cutoff level for discriminating between the DAD group and the other groups. Statistical analysis was performed using a statistical software package (SAS 9.1.3, SAS Institute, Cary, NC, USA). A P value <0.05 was considered statistically significant.

## Results

### Interpretation of chest HRCT images

The interobserver variability before reaching consensus agreement was good (Kappa value = 0.81).

### Comparison of various parameters according to HRCT patterns

Table 1 shows the patient characteristics and categories of causal drugs among patients according to each HRCT pattern. There were four patients with the OP pattern, 24 with the NSIP pattern, 11 with the HP pattern, and eight with the DAD pattern. There were no significant differences in the duration from HRCT chest scanning to the serum sample collection and in causal drugs among the patient groups. In the DAD group, none of the patients improved without glucocorticoid administration, whereas 75.0% (three of four patients) of the OP group, 45.8% (11 of 24 patients) of the NSIP group, and 36.4% (four of 11 patients) of the HP group improved without glucocorticoid administration. In the DAD group, 37.5% of the patients (three of eight patients) underwent mechanical ventilation, whereas none of the patients in the other groups required mechanical ventilation. In the DAD group, the 30-day mortality rate was 37.5% (three of eight patients). In contrast, none of the patients in the other groups died within 30 days.

**Table 1:** Comparison of patient characteristics according to each HRCT pattern

	**OP**		**NSIP**		**HP**		**DAD**		***P*** **value**
	**(N = 4)**		**(N = 24)**		**(N = 11)**		**(N = 8)**		
Age (years)	71.5	(68-77)	68.0	(34-84)	64.0	(48-79)	69.5	(62-80)	N.S.
Sex (male/female)	2/2		18/6		5/6		5/3		N.S.
Smoking (s/ex/n)	0/3/1		2/16/6		0/6/5		3/3/2		N.S.
Duration from HRCT chest scanning to the serum sample collection (days)	1	(1–3)	4.5	(0–21)	3	(0–16)	2	(0–20)	N.S.
Causal drugs									N.S.
Cytotoxic agents	0		8		1		2		
DMARDs	1		4		3		0		
Chinese herbal medicine	1		1		0		4		
Antineoplastic agent	0		3		2		0		
NSAIDs	1		2		0		1		
Antibiotics	1		0		2		0		
Interferon	0		1		1		0		
Others	0		5		2		1		
Improved without GC administration (%)	75.0		45.8		36.4		0*		0.05
Ventilator needed (%)	0.0		0.0		0.0		37.5#		0.001
Mortality rate at 30 days (%)	0.0		0.0		0.0		37.5#		0.001

Data presented as median (range).

N = number of patients; OP = organizing pneumonia pattern; NSIP = nonspecific interstitial pneumonia pattern; HP = hypersensitivity pneumonitis pattern; DAD = diffuse alveolar damage pattern; N.S. = not significant; s/ex/n = current smoker/ex-smoker/nonsmoker; HRCT = high-resolution computed tomographic; DMARDs = Disease-modifying anti-rheumatic drugs; NSAIDs = non-steroidal anti-inflammatory drugs; GC = glucocorticoid. *; p < 0.01 compared with OP group. p < 0.05 compared with NSIP group. #; p < 0.01 compared with NSIP group. p < 0.05 compared with HP group.

Table 2 lists laboratory data of the patients according to each HRCT pattern. Peripheral white blood cell counts were significantly higher in the DAD group than in the NSIP and HP groups. Serum HSP47 levels were significantly higher in the DAD group than in the OP, NSIP, and HP groups. The P/F ratios were significantly lower and the A-a DO_2_ was significantly higher in the DAD group than in the OP, NSIP, and HP groups. Serum KL-6 and SP-A levels were significantly different among the groups, but pair-wise comparison by post-hoc analysis did not show any significant difference in these values between any pair of groups. Serum SP-D levels and BAL fluid findings were not significantly different among the patient groups. Serum HSP47 levels in the OP, NSIP, and HP groups did not differ significantly compared with those in healthy controls (median, 501.6 [range, 149.0-1222.5] pg/mL), while serum HSP47 levels in the DAD group were significantly higher than those in healthy controls (P <0.01).

**Table 2:** Comparison of various parameters according to each HRCT patterns

	**OP**			**NSIP**			**HP**			**DAD**			***P*** **value**
	**(N = 4)**			**(N = 24)**			**(N = 11)**			**(N = 8)**			
			[n]			[n]			[n]			[n]	
Hematologic data													
WBC (/mm^3^)	10600	(4300-14600)	[4]	6400	(3500-11200)	[23]	6700	(3500-13400)	[11]	10450#	(4900-20800)	[8]	0.01
Hb (g/dL)	10.6	(7.9-13.6)	[4]	11.7	(8.5-16.2)	[23]	12.0	(8.6-14.4)	[11]	11.4	(8.6-12.2)	[8]	N.S.
Plt (×10^4^/mm^3^)	25.4	(17.8-31.1)	[4]	22.6	(9.7-45.5)	[23]	26.3	(12.5-33.1)	[11]	24.6	(8.9-46.9)	[8]	N.S.
Serum markers													
KL-6 (U/mL)	293.0	(144-311)	[3]	902.0	(320-3884)	[22]	518.5	(264-3697)	[10]	442.0	(147-2218)	[7]	0.03§
SP-A (ng/mL)	48.5	(42.6-54.4)	[2]	50.5	(35.9-219)	[19]	98.5	(47.1-310)	[10]	115.0	(43.4-169)	[5]	0.04§
SP-D (ng/mL)	40.0	(33-169)	[3]	193.0	(23-753)	[20]	186.5	(56.2-532)	[10]	168.0	(60.0-462)	[7]	N.S.
HSP47 (pg/mL)	301.1	(100.5-985.7)	[4]	554.4	(61.9-1356)	[24]	847.9	(347.7-2012)	[11]	1868.3*	(816.3-2843.9)	[8]	<0.001
Respiratory parameters			[4]			[21]			[11]			[8]	
P/F ratio (mmHg)	397.9	(370.9-475.2)		393.8	(260-523.8)		355.7	(83.1-542.9)		150.7*	(71-293.8)		<0.001
A-a DO_2_ (mmHg)	13.5	(4.3-32.3)		18.9	(−11.3-69.1)		36.3	(−9.5-450.0)		214.8*	(42.4-474.7)		<0.001
BAL fluid findings			[4]			[22]			[9]			[5]	
Total cell count (× 10^5^/mL)	6.29	(3.6-27.2)		4.66	(1.44-54.9)		9.01	(1.8-15.5)		5.28	(1.27-8.9)		N.S.
Macrophages (%)	57.2	(28.9-73.6)		57.9	(4.6-85.6)		33.8	(4.2-82.7)		32.8	(4.0-94.8)		N.S.
Lymphocytes (%)	29.6	(22.9-52.9)		26.6	(1.7-77.8)		54.8	(13.7-95.4)		31.3	(1.9-47)		N.S.
Neutrophils (%)	3.8	(0.6-6.9)		2.5	(0-93.6)		3.5	(0-15.8)		32.1	(0.4-52)		N.S.
Eosinophils (%)	0.8	(0.5-29.9)		2.7	(0-50.9)		1.6	(0-15.5)		2.0	(0-2.88)		N.S.
CD4/8 ratio	1.5	(0.36-2.22)		1.8	(0.14-7.5)		0.83	(0.14-10.4)		1.08	(0.4-1.4)		N.S.

Data are presented as medians (range). N = number of patients; n = number of patients examined; OP = organizing pneumonia pattern; NSIP = nonspecific interstitial pneumonia pattern; HP = hypersensitivity pneumonitis pattern; DAD = diffuse alveolar damage pattern; N.S. = not significant; WBC = white blood cell count; Hb = hemoglobin; Plt = platelet count; KL-6 = Krebs von den Lungen-6; SP-A = surfactant protein-A; SP-D = surfactant protein-D; HSP47 = heat shock protein 47; P/F ratio = PaO_2_/fraction of inspired oxygen ratio; A-a DO_2_ = alveolar-arterial difference of oxygen; BAL = bronchoalveolar lavage; CD4/8 ratio = CD4/CD8 ratio of lymphocyte subsets. #; p < 0.01 compared with NSIP and HP group. *; p < 0.01 compared with OP, NSIP, and HP group. §; Significantly different among the groups; however, pair-wise comparison by post-hoc analysis did not show any significant difference between any pairs.

### Comparison of various parameters according to the need for glucocorticoid administration

Eighteen of 47 patients enrolled in this study improved without glucocorticoid administration. The other 29 patients required glucocorticoid administration. Each attending physician determined the indication for glucocorticoids based on the patient’s status, and these physicians were not aware of the results of serum HSP47 level testing. All patients in the DAD group required glucocorticoid administration. The P/F ratio was significantly lower and the A-a DO_2_ was significantly higher in the group of patients requiring glucocorticoids when compared with patients that did not require glucocorticoids. Serum HSP47 and SP-A levels were significantly higher in the group of patients requiring glucocorticoids when compared with patients that did not require glucocorticoids. In contrast, there was no difference in serum KL-6 or SP-D levels or in BAL fluid findings when comparing the group of patients requiring glucocorticoids with the patients that did not require glucocorticoids (Table 3).

**Table 3:** Comparison of various parameters according to the need for glucocorticoid administration

	**Improved without glucocorticoid**			**Glucocorticoid needed**			***P*** **value**
	**(N = 18)**			**(N = 29)**			
			[n]			[n]	
Age (years)	68.5	(34-84)	[18]	68.0	(36-80)	[29]	N.S.
Sex (male/female)	13/5		[18]	17/12		[29]	N.S.
Smoking (s/ex/n)	1/12/5		[18]	4/16/9		[29]	N.S.
HRCT patterns			[18]			[29]	0.05
OP	3			1			
NSIP	11			13			
HP	4			7			
DAD	0			8			
Mortality rate at 30 days (%)	0.0			10.3			N.S.
Hematologic data							
WBC (/mm^3^)	6300	(3500-14600)	[18]	7400	(3500-20800)	[28]	N.S.
Hb (g/dL)	12.3	(8.5-14.3)	[18]	10.9	(7.9-16.2)	[28]	N.S.
Plt (×10^4^/mm^3^)	21.5	(12.5-34.1)	[18]	25.3	(8.9-46.9)	[28]	N.S.
Serum markers							
KL-6 (U/mL)	601.0	(264-2115)	[16]	531.5	(144-3884)	[26]	N.S.
SP-A (ng/mL)	52.0	(35.9-141)	[15]	89.8	(43.2-310)	[21]	0.02
SP-D (ng/mL)	158.0	(23-653)	[15]	175.0	(33-753)	[25]	N.S.
HSP47 (pg/mL)	460.2	(61.9-1631)	[18]	1023.0	(100.5-2843.9)	[29]	0.003
Respiratory parameters			[17]			[27]	
P/F ratio (mmHg)	409.0	(260-542.8)		307.3	(71-523.8)		0.005
A-a DO_2_ (mm/Hg)	15.9	(-9.5-69.1)		37.8	(-11.4-474.7)		0.007
BAL fluid findings			[16]			[24]	
Total cell count (×10^5^/mL)	4.4	(1.44-15.5)		5.4	(1.27-54.9)		N.S.
Macrophages (%)	65.3	(4.3-85.4)		37.3	(4-94.8)		N.S.
Lymphocytes (%)	26.6	(7.7-95.3)		34.8	(1.7-82)		N.S.
Neutrophils (%)	3.2	(0-13.6)		3.0	(0-93.6)		N.S.
Eosinophils (%)	2.1	(0-29.9)		1.8	(0-50.8)		N.S.
CD4/8 ratio	0.91	(0.14-7.05)		1.59	(0.14-10.4)		N.S.

Data are presented as medians (range). HRCT = high-resolution computed tomographic chest scanning. For other abbreviations, see Tables 1 and 2.

### Comparison of various parameters according to survival at 30 days

All patients who died within 30 days had the DAD pattern on HRCT. The P/F ratios were significantly lower and the A-a DO_2_ was significantly higher in nonsurvivors than in survivors. Serum HSP47 levels tended to be higher in nonsurvivors than in survivors, but this difference did not reach the level of statistical significance (p = 0.08). Serum KL-6, SP-A, and SP-D levels and BAL fluid findings did not differ significantly when comparing nonsurvivors and survivors (Table 4).

**Table 4:** Comparison of various parameters according to survival at 30 days

	**Survivors**			**Nonsurvivors**			***P*** **value**
	**(N = 44)**			**(N = 3)**			
			[n]			[n]	
Age (years)	68.0	(34-84)	[44]	69.0	(62-71)	[3]	N.S.
Sex (male/female)	28/16		[44]	2/1		[3]	N.S.
Smoking (s/ex/n)	4/26/14		[44]	1/2/0		[3]	N.S.
HRCT patterns			[44]			[3]	0.001
OP	4			0			
NSIP	24			0			
HP	11			0			
DAD	5			3			
Hematologic data							
WBC (/mm^3^)	6700.0	(3500-14600)	[43]	16500.0	(4900-20800)	[3]	N.S.
Hb (g/dL)	11.6	(7.9-16.2)	[43]	11.2	(8.6-12.2)	[3]	N.S.
Plt (×10^4^/mm^3^)	24.6	(9.7-46.9)	[43]	16.6	(8.9-40.4)	[3]	N.S.
Serum markers							
KL-6 (U/mL)	502.0	(144-3884)	[40]	1544.0	(870-2218)	[2]	N.S.
SP-A (ng/mL)	64.6	(35.9-310)	[34]	65.5	(43.4-87.6)	[2]	N.S.
SP-D (ng/mL)	168.5	(23-753)	[38]	315.0	(168-462)	[2]	N.S.
HSP47 (pg/mL)	773.5	(61.9-2500.8)	[44]	1862.4	(816.3-2843.9)	[3]	N.S.
Respiratory parameters			[41]			[3]	
P/F ratio (mmHg)	372.4	(71-542.8)		179.3	(122.2-233.2)		0.03
A-a DO_2_ (mmHg)	24.1	(−11.4-474.7)		168.8	(88.6-258)		0.03
BAL fluid findings			[38]			[2]	
Total cell count (×10^5^/mL)	5.3	(1.4-54.9)		3.28	(1.27-5.28)		N.S.
Macrophages (%)	43.4	(4.0-85.6)		65.3	(32.7-97.8)		N.S.
Lymphocytes (%)	34.5	(1.7-95.3)		16.6	(1.9-31.3)		N.S.
Neutrophils (%)	3	(0-93.6)		17.3	(0.4-34.3)		N.S.
Eosinophils (%)	2	(0-50.8)		2.22	(1.55-2.88)		N.S.
CD4/8 ratio	1.42	(0.14-10.4)		0.58	(0.40-0.76)		N.S.

Data are presented as medians (range). For abbreviations, see Tables 1, 2, and 3.

### Receiver operating characteristic curve

**Figure 1:**
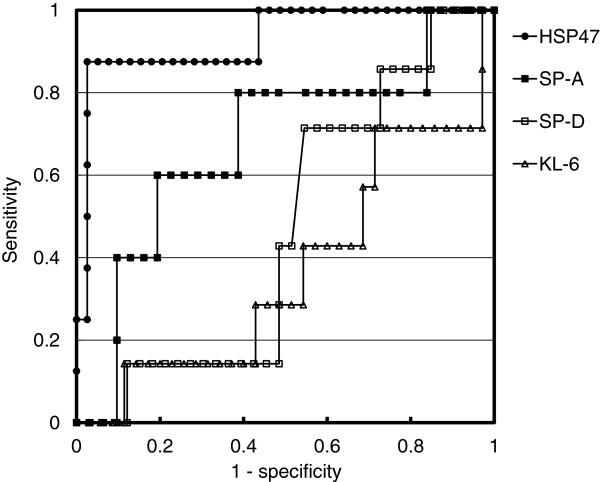
**Receiver operating characteristic curve.** Based on a receiver operating characteristic curve, the cut-off level of heat shock protein (HSP) 47 that resulted in the highest diagnostic accuracy for drug-induced lung diseases (DILD) with a diffuse alveolar damage (DAD) pattern was 1711.5 pg/mL. This value discriminated between the DAD group and the other groups with 87.5% sensitivity and 97.4% specificity and was associated with a diagnostic accuracy of 95.7%. Use of serum HSP47 levels for diagnosis of DILD with a DAD pattern resulted in the largest area under the curve (0.929) when compared with the use of serum Krebs von den Lungen-6 (KL-6), surfactant protein (SP)-A, and SP-D levels (0.367, 0.677, and 0.468, respectively).

Based on a receiver operating characteristic curve (Figure 1), the cut-off level of HSP47 that resulted in the highest diagnostic accuracy for DILD with a DAD pattern was 1711.5 pg/mL. This value discriminated between the DAD group and the other groups with 87.5% sensitivity and 97.4% specificity and was associated with a diagnostic accuracy of 95.7%. Use of serum HSP47 levels for diagnosis of DILD with a DAD pattern resulted in the largest area under the curve (0.929) when compared with the use of serum KL-6, SP-A, and SP-D levels (0.367, 0.677, and 0.468, respectively).

### Correlations between serum HSP47, KL-6, SP-A, and SP-D levels and respiratory parameters

**Figure 2:**
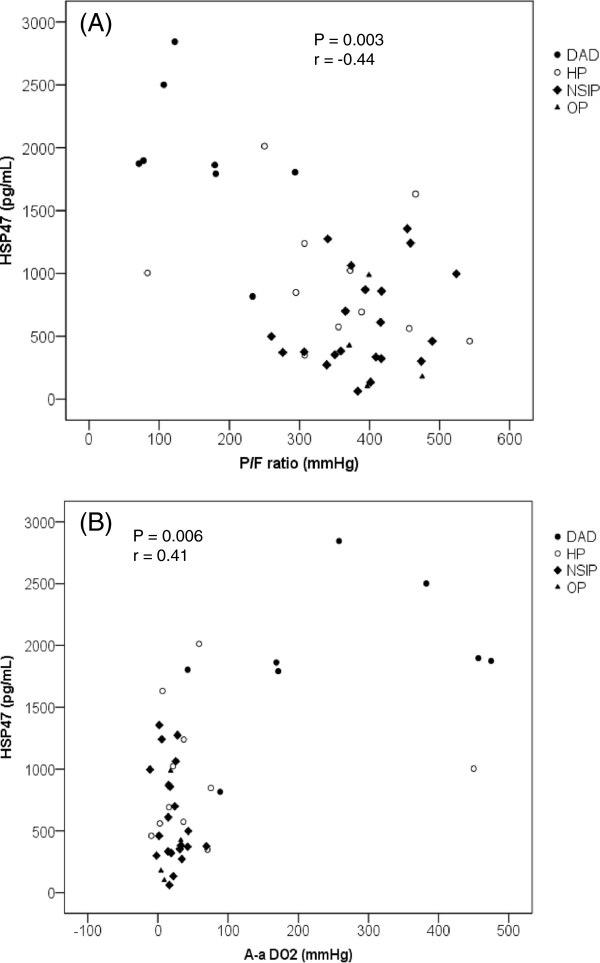
**Correlation between serum heat shock protein (HSP) 47 levels and respiratory parameters.** Significant correlation was demonstrated between serum HSP47 levels and **(A)** PaO_2_/fraction of inspired oxygen ratio (P/F ratio) and **(B)** alveolar-arterial difference of oxygen (A-a DO_2_). Each dot represents a patient with a diffuse alveolar damage (DAD) (closed circle), hypersensitivity pneumonitis (HP) (open circle), nonspecific interstitial pneumonia (NSIP) (quarry), or organizing pneumonia (OP) (triangle) pattern.

A significant correlation was demonstrated between serum HSP47 levels and the P/F ratio and A-a DO_2_ (Figure 2). In contrast, serum KL-6, SP-A, or SP-D levels did not correlate with the P/F ratio or with A-a DO_2_ (data not shown).

### Histopathological and immunohistochemical findings

**Figure 3:**
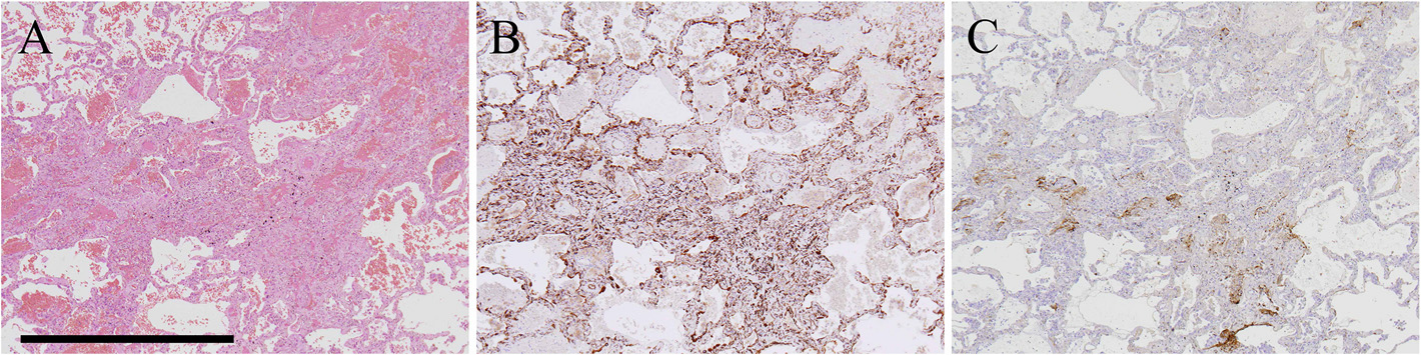
**Histopathological and immunohistochemical findings.** Photomicrographs of histological and immunohistochemical studies from representative diffuse alveolar damage (DAD) autopsy specimens are shown. This DAD patient had a final diagnosis of drug-induced lung disease (DILD). Expression of HSP47 was noted predominantly in fibroblasts, epithelial cells, and endothelial cells. **A**; hematoxylin-eosin staining. **B**; heat shock protein (HSP) 47. **C**; type I procollagen. Scale bar = 1 mm.

Photomicrographs of histological and immunohistochemical studies from representative DAD autopsy specimens are shown in Figure 3. This DAD patient had a final diagnosis of DILD. The chest HRCT scanning of this patient at the time of hospitalization revealed a DAD pattern. The serum HSP47 level was 2843.9 pg/mL at that time. Histopathological examination revealed diffuse involvement, including hyaline membranes, interstitial edema and inflammation, and severe fibrosis. Expression of HSP47 and type I procollagen was diffuse and high. Expression of HSP47 was noted predominantly in fibroblasts, epithelial cells, and endothelial cells. Negative control studies using non-specific immunoglobulin-G revealed no positive cells (data not shown).

## Discussion

This is the first study to investigate the utility of screening serum HSP47 in the management of DILD. We demonstrated that serum HSP47 levels were elevated in patients with DILD with a DAD pattern who had the worst outcomes among the different subgroups. All patients with a DAD pattern in HRCT required glucocorticoid administration, and all patients who underwent mechanical ventilation or died within 30 days had the DAD pattern in HRCT. In combination with findings from previous studies [1,2], the present data suggest that the DAD pattern in HRCT is an important prognostic factor in patients with DILD. HSP47 was superior to KL-6, SP-A, and SP-D for discriminating between the DAD and other groups. The present study suggested that HSP47 could be a useful marker to identify patients with DILD with a DAD pattern. This study also showed that serum HSP47 levels significantly correlated with various respiratory parameters. In addition, serum levels of HSP47 were significantly higher in the group of patients requiring glucocorticoids than in patients who did not require glucocorticoids. Serum levels of HSP47 also tended to be higher in nonsurvivors than in survivors. Unlike most molecular chaperones that recognize a wide variety of target proteins, the only substrate protein for HSP47 is collagen [12]. Hence, the increase of HSP47 might be a specific reflection of pulmonary fibrotic activity in patients with DILD. Irrespective of the primary disease, upregulation of HSP47 is a common phenomenon during the fibrotic process of any organ [8-15,17-19]. Therefore, it is likely that assessment of serum HSP47 levels might help to define those patients at risk of developing fibrotic complications and to monitor the response to treatment.

We previously reported that only a few cells positive for HSP47 were detected in control lungs, while the expression of HSP47 was increased in idiopathic usual interstitial pneumonia specimens [17,24]. However, we also reported that serum levels of HSP47 did not differ significantly between patients with stable IPF and healthy controls [22], while HSP47 serum levels were markedly elevated in patients with acute exacerbations of IPF [19]. The present study demonstrated that serum HSP47 levels were elevated in patients with DILD with a DAD pattern, while serum HSP47 levels in patients with other patterns were similar to those in healthy controls. Taken together, serum HSP47 levels could be elevated specifically in patients with DAD. Serum HSP47 could be useful for the specific identification of patients with DILD with a DAD pattern who are at high risk of poor outcomes.

We recently reported that the expression of HSP47 in autopsied DAD lung specimens of patients with acute exacerbation of IPF was greater than that in usual interstitial pneumonia lung specimens of patients with stable IPF [19]. The present study also demonstrated that the expression of HSP47 was also markedly increased in DAD autopsied lung specimens of patients with DILD. The specific elevation of serum HSP47 in DILD with a DAD pattern might be due to not only the strong expression of HSP47 in lung tissues but also the distinctive characteristics of DAD, including severe inflammation, tissue destruction, apoptosis and/or cell necrosis, and increased vascular permeability [25]. These changes might induce leakage of HSP47 protein into the peripheral blood.

The histopathologic pattern of DAD is also seen in the other types of lung injury, such as acute respiratory distress syndrome, acute interstitial pneumonia, connective tissue disease-associated interstitial pneumonia, and so on. It is possible that the expression of HSP47 could be upregulated in DAD lung tissue independent of the specific disease. Hence, serum HSP47 levels could be a useful marker for other fatal and rapidly progressive fibrotic lung diseases that have histological manifestations similar to those of DAD.

Bronchoscopy with BAL is frequently performed in patients with DILD. In such patients, BAL fluid cell counts are usually elevated; lymphocytosis, neutrophilia, or rarely, eosinophilia may be seen [26]. In this study, neither the pattern of cellularity nor any other findings differed when comparing subgroups of patients with DILD. Therefore, the main role of bronchoscopy in this context appears to be the exclusion of infection or recurrent primary illness.

Treatment for DILD is largely supportive and consists of immediate causative drug discontinuation, administration of supplemental oxygen, empiric antibiotics, and mechanical ventilation as clinically indicated. Systemic glucocorticoids are usually recommended, although the evidence to support their use is largely anecdotal, and fatalities still occur despite empiric treatment with high-dose glucocorticoids. HSP47 is involved in the molecular maturation of collagens, and selective blockade of HSP47 activity in fibrotic diseases is a potentially novel therapy. Previous studies in animal models of fibrosis (including pulmonary fibrosis) have shown that down-regulation of HSP47 expression by antisense oligodeoxynucleotides or by small interfering ribonucleic acid reduces collagen production and subsequently diminishes progression of fibrosis [16,27-29]. Thus, downregulation of HSP47 might delay or diminish the progression of fibrosis by reducing the accumulation of collagen. This strategy might be useful in the management of patients with DILD or other fibrotic diseases.

Some limitations to this study should be noted. First, the number of patients enrolled was small, and pulmonary toxicities were caused by many different kinds of drugs. An analysis of a large number of patients with DILD caused by a single drug would strengthen the evidence. Moreover, it should be noted that the number of nonsurvivors (n = 3) was too small to draw conclusions as to whether HSP47 is a useful prognostic marker for patients with DILD. Second, the present study was based on the classification of images in the absence of pathological evidence. Third, this was a retrospective study, and the indication for glucocorticoid administration was determined by attending physicians who were not aware of the results of serum HSP47 level testing. Fourth, sequential changes in HSP47 serum levels after the onset of DILD could not be analyzed due to the lack of sufficient number of serum stocks. Therefore, it is not yet clear whether evaluation of these sequential changes is useful to monitor disease progression and response to treatment. A prospective multicenter study with a larger patient cohort is planned in order to overcome the above-mentioned limitations of the present study.

## Conclusions

In conclusion, this study demonstrated that serum HSP47 levels were elevated in patients with DILD with a DAD pattern who had the worst outcomes among the different subgroups, and that this was correlated with P/F ratio and A-a DO_2_. Further studies involving larger cohorts of patients are warranted to elucidate whether HSP47 is a useful prognostic marker for patients with DILD.

## Abbreviations

A-a DO2: Alveolar-arterial difference of oxygen; BAL: Bronchoalveolar lavage; DAD: Diffuse alveolar damage; DILD: Drug-induced lung disease; ELISA: Enzyme-linked immunosorbent assay; FiO2: Fraction of inspired oxygen; HP: Hypersensitivity pneumonitis; HRCT: High-resolution computed tomographic; HSP47: Heat shock protein 47; IPF: Idiopathic pulmonary fibrosis; KL-6: Krebs von den Lungen-6; NSIP: Nonspecific interstitial pneumonia; OP: Organizing pneumonia; P/F ratio: PaO_2_/fraction of inspired oxygen (FiO_2_) ratio; SP: Surfactant protein.

## Competing interests

T. Kakugawa received a research grant from Takeda Science Foundation and the Kato Memorial Trust for Nambyo Research. T. Kakugawa and S. Yokota have a patent application pending for research related to this manuscript. S. Kohno reports grants and personal fees from Astellas Pharma Inc., grants and personal fees from Daiichi Sankyo Co., Ltd., grants and personal fees from Dainippon Sumitomo Pharma, grants and personal fees from Pfizer, Inc., grants and personal fees from Shionogi & Co., Ltd., grants from Astrazeneca KK, grants from Eisai Co., Ltd., grants from Bayer Yakuhin, Ltd., grants from Taisho Toyama Pharmaceutical Co., Ltd., personal fees from MSD K.K., grants from Kyorin Pharmaceutical Co., Ltd., grants from Meiji Seika Pharma Co., Ltd., grants from Takeda Pharmaceutical Co., Ltd., grants from Chugai Pharmaceutical Co., Ltd., grants from Otsuka Pharmaceutical Co., Ltd., grants from Ono Pharmaceutical Co., Ltd., grants from TAIHO Phamaceutical Co., Ltd., outside the submitted work.

## Authors’ contributions

TK made substantial contributions to study conception and design. TK, NS, SH, SN and YI collected clinical samples. SY helped determine serum levels of HSP47 by ELISA. HK and YM helped prepare recombinant HSP47 protein. TK helped perform immunohistochemistry. TK and TH performed pathological assessments. TK and MM were involved in statistical analysis. TK drafted the article. SY, YI, NS, HM, KN, and SK critically revised the article for important intellectual content. All authors read and approved the final manuscript.

## Authors’ information

Yasuhiro Matsuoka, Present address: Research Laboratories, Research & Development Div., Kyowa Medex Co., Ltd., Shizuoka, Japan.
